# Fenofibrate Exerts Antitumor Effects in Colon Cancer via Regulation of DNMT1 and CDKN2A

**DOI:** 10.1155/2021/6663782

**Published:** 2021-04-16

**Authors:** Rui Kong, Nan Wang, Wei Han, Wen Bao, Jie Lu

**Affiliations:** Department of Gastroenterology, Shanghai Tenth People's Hospital Affiliated to Tongji University, Tongji University, School of Medicine, Shanghai 200072, China

## Abstract

Peroxisome proliferator-activated receptor alpha (PPARA) is the molecular target of fibrates commonly used to treat dyslipidemia and diabetes. Recently, the potential role of PPARA in other pathological conditions, such as cancers, has been recognized. Here, using bioinformatics analysis, we found that PPARA was expressed at relatively low levels in pancancers, and Kaplan-Meier analyses revealed that high PPARA protein expression was correlated with better survival of patients with colon cancer. *In vitro* experiments showed that fenofibrate regulated cell cycle distribution, promoted apoptosis, and suppressed cell proliferation and epithelial mesenchymal transition by activating PPARA. PPARA activation inhibited DNMT1 activity and abolished methylation-mediated CDKN2A repression. Downregulation of cyclin-CDK complexes led to the restoration of CDKN2A, which caused cell cycle arrest in the G1 phase via regulation of the CDKN2A/RB/E2F pathway. Finally, we demonstrated that fenofibrate administration inhibited tumor growth and DNMT1 activity *in vivo*. The PPARA agonist, fenofibrate, might serve as an applicable agent for epigenetic therapy of colon cancer patients.

## 1. Introduction

Colorectal cancer (CRC) ranks third in terms of morbidity and fourth in terms of mortality. CRC is also the most common type of cancer worldwide, with almost 900,000 deaths each year [1, 2]. Although new treatment options, including immunotherapy and neoadjuvant chemotherapy, have significantly improved patient prognosis, the 5-year survival rate of CRC remains below 15% [3]. Thus, investigations of the molecular mechanisms involved in cancer initiation are necessary to develop new therapeutic strategies.

In addition to genetic alterations (i.e., deletion, amplification, and translocation), epigenetic modifications play an important role in malignant progression of tumors. CpG islands (CGI) are contiguous groups of dinucleotides mainly located at the 5′ end of a gene and are characterized by high GC content [4]. Most CGIs in gene promoters are unmethylated, allowing active transcription [5]. CGI methylation changes are hallmarks of many human cancers and lead to concomitant gene inactivation [6–8]. DNA methyltransferase 1 (DNMT1) is a major DNA methyltransferase responsible for methylation maintenance during DNA replication, and inactivation of DNMT1 in mice results in early embryonic lethality [9]. DNMT3a and DNMT3b mainly act as de novo methyltransferases [10].

CRCs are characterized by lower levels of absolute genomic methylation compared with normal tissues, a characteristic that contributes to high genomic instability and results in cancer development [11]. In addition, promoter hypermethylation of specific genes has been identified in CRCs, as well as methylated CGIs that are associated with gene silencing. Hypermethylation in several tumor suppressor genes such as RASSF1, PTEN, and CDKN2A is associated with abnormal cellular activities, including aberrant cell aging, proliferation, and death [12–14].

Peroxisome proliferator-activated receptor alpha (PPARA) is a ligand-activated transcription factor that belongs to the nuclear hormone receptor superfamily [15, 16]. Studies have demonstrated that PPARA plays a critical role in lipid metabolism and the inflammatory response [17, 18]. Fenofibrate is a selective PPARA agonist that regulates lipid transport and metabolism, and is widely used in the treatment of hyperlipidemia [19]. In addition to the metabolic efficacy, recent studies have revealed the antitumor function of PPARs [20–24]. Studies have also confirmed the oncosuppressive effect of fenofibrate in various human cancer cell lines through different signaling pathways [25–27]. Reports also revealed the inhibitory role of PPARA on DNMT1 activity in mouse models [28]. In the present study, we demonstrated that the PPARA agonist, fenofibrate, inhibited DNMT1-mediated methylation of CDKN2A and exerted anticancer effects by promoting cell apoptosis, inhibiting cell migration, and suppressing cell proliferation via the CDKN2A/RB/E2F pathway.

## 2. Materials and Methods

### 2.1. Cell Culture

The colorectal cancer cell line HCT116, the colon cancer cell lines SW480 and Caco-2, and the two normal intestine epithelial cell lines NCM460 and HIEC were obtained from Chinese Academy of Sciences Committee Type Culture Collection Cell Bank (Beijing, China). HCT116 was cultured in complete DMEM (Thermo Fisher Scientific, Waltham, MA), and the other cell lines were maintained in RPMI 1640 (Thermo Fisher Scientific, Waltham, MA) supplemented with 10% fetal bovine serum (FBS, Thermo Fisher Scientific, Waltham, MA) and 1% penicillin-streptomycin.

Inhibition of DNMT1 activity was conducted using 5 *μ*M 5-azacytidine (MedChemExpress, Shanghai, China), and the treatment time was 24 h. The pcDNA DNMT1 was transfected into HCT116 and SW480 cells for upregulation of DNMT1 expression. The pcDNA DNMT1 were synthesized by GenePharma Co. Ltd. (Shanghai, China).

Cells were exposed to different concentrations of fenofibrate (Topscience Co., Ltd., Shanghai, China) for 48 hours, and morphology changes were observed using a phase-contrast microscope and imaged (200x).

### 2.2. Cell Viability Assay

Colon cancer cell lines SW480 and the colorectal cancer cell line HCT116 were plated at a density of 5000 cells/mL in 96-well plates (100 *μ*L medium per well) with three replicates. Cells were treated with fenofibrate in the pharmacologic concentration range 0–260 *μ*M for 24 h and 48 h. Cell viability was detected using the cell counting kit (YEASEN, Shanghai, China) according to the instructions.

### 2.3. Colony Formation Assay

Cells were trypsinized, counted, and seeded in a 6-well plate at 700 cells per well, treated with different concentrations of fenofibrate. After 14 days, the visible colonies were counted and photographed.

### 2.4. Wound Healing Assay

HCT116 and SW480 cells were grown on 6-well plates. Scratch was made using a 200 *μ*L pipette tip when the cell confluence reached 80%-90%. After scratching, cells were washed with phosphate-buffered saline and then cultured in serum-free medium. The healing rate was quantified with measurements of the gap size after the culture using ImageJ software.

### 2.5. Cell Immunofluorescence

HCT116 and SW480 cells were seeded in 6-well culture plates plated with cell climbing slices. After being treated with fenofibrate for 24 h, cells were fixed with 4% paraformaldehyde and permeabilized with 0.1% Triton X-100. Then, cells were incubated with primary antibodies (E-cadherin, vimentin, PCNA). The chromosomes were counter-stained with DAPI (Beyotime, Shanghai, China). Images were viewed with a fluorescent microscope.

Apoptosis was analyzed by means of TUNEL assay using the EdUTP TUNEL cell detection kit (Beyotime, Shanghai, China) according to the manufacturer's instructions. Photomicrographs were taken under confocal microscopy.

### 2.6. EDU Staining

5-Ethynyl-2′-deoxyuridine (EdU) staining assay was carried out on fenofibrate-treated cells utilizing an EdU immunofluorescence staining kit (Ribobio, China) according to the manufacturer's instructions [29]. The results were observed using an inverted fluorescence microscope (200x).

### 2.7. Flow Cytometry Analysis

HCT116 and SW480 cell lines were treated with various doses of fenofibrate for 24 h, then stained with annexin V FITC and propidium iodide (PI) (BD Biosciences, San Jose, CA). Cell apoptosis was analyzed by flow cytometry (Cytomics FC500; Beckman Coulter, Fullerton, CA).

The distribution of cell cycle phases was analyzed by the cell cycle detection kit (Beyotime, China). Samples were fixed with 75% ethanol at −20°C for 24 h. The fixed cells were treated with RNaseA and stained with propidium iodide following the manufacturer's instruction [30]. The cell cycle analysis was studied by flow cytometry.

### 2.8. Animal Experiments

Animal experiments were performed according to the National Institutes of Health Guidelines for the Care and Use of Laboratory Animals and were approved by the Animal Care and Use Committee of Shanghai Tongji University, China. The nude mice were randomly divided into 2 groups (*n* = 6), and each mouse was subcutaneously injected with 2 × 10^7^ HCT116 cells in the right axilla. Fenofibrate was suspended in saline and intragastrically administered at 200 mg/kg per mouse once a day. Mice were anesthetized and sacrificed three weeks after fenofibrate administration. Tumor size and mouse weight were recorded during the experiment. Tissues were harvested for further analysis.

Xenografted tissues were fixed with 4% paraformaldehyde and embedded in paraffin, sectioned, and stained with hematoxylin and eosin. Histopathological changes were observed by microscopy (200x).

### 2.9. DNMT1 Content

The measurement of DNMT1 content in fenofibrate-treated cells or tumor tissues was conducted using Human or Mouse DNMT1 ELISA kit (HZBIO, Shanghai, China). Samples were added to each well in enzyme-labeled coated plates and incubated at 37°C for 30 minutes. After washing, coloration was developed using chromogen reagents A and B; then, the reaction was terminated by stop solution. The absorbance value was measured at 450 nm wavelength using a microplate reader (BioTek microplate reader).

### 2.10. Methylation-Specific PCR

Genomic DNA was extracted using a genomic DNA extraction kit (TIANGEN). Eluted DNA (20 *μ*L) was subjected to bisulfite modification using the EpiTect Fast Bisulfite Conversion Kit (Qiagen). Methylation status of CDKN2A promoter was analyzed using the methylation-specific primers (M) and nonmethylation-specific primers (U). The amplification products were separated by agarose gel electrophoresis and visualized by SYBR Green staining under UV light. Primers (Table S1) used in methylation-specific PCR (MSP) were the same as the sequences described by Herman et al. [31].

### 2.11. Bioinformatics Analysis of Human Tumor Samples from TCGA Dataset

RNA sequencing profiles and relevant clinical information of pancancer samples were retrieved from The Cancer Genome Atlas (TCGA) data portal in October, 2020. RNA-seq data was normalized by fragments per kilobase per million (FPKM) using log2 scale. Transcription levels of DNMT1, DNMT3a, DNMT3b, PPARs, ACOX1, and CDKN2A were analyzed. Survival probabilities were computed by the Kaplan-Meier method. In addition, Cox regression analysis was conducted to calculate the hazard ratios of genes of interest in multiple cancer types. Moreover, the correlation between clinical stage and gene expression was evaluated via “ggpubr” package in R.

### 2.12. Western Blot Analysis

Total protein from cells or tissues was extracted using RIPA lysate (Invitrogen, USA). Equal amount of protein samples (40 *μ*g for cell samples per lane and 80 *μ*g for tissue samples per lane) was run on sodium dodecylsulfate-polyacrylamide (SDS) gel and then transferred to polyvinylidene difluoride (PVDF) membranes. Membranes were blocked with 5% nonfat milk for 1 h and then incubated overnight at 4°C with primary antibodies. Anti-DNMT1 (1 : 500, Abcam, Cambridge, UK), anti-PPARA (1 : 1000, Abcam, Cambridge, UK), anti-E2F1 (1 : 500, Cell Signaling Technology, Danvers, MA), anti-pRb (1 : 500, Cell Signaling Technology, Danvers, MA), anti-CDKN2A (1 : 500, Cell Signaling Technology, Danvers, MA), anti-CyclinD1 (1 : 1000, Cell Signaling Technology, Danvers, MA), anti-CDK4 (1 : 1000, Cell Signaling Technology, Danvers, MA), anti-CDK6 (1 : 1000, Cell Signaling Technology, Danvers, MA), and anti-RB (1 : 200, Santa Cruz Biotechnology, CA) antibodies were used in this experiment. Then, the PVDF membranes were incubated with corresponding secondary antibodies at room temperature for 1 h. Primary and secondary antibody diluent for WB was used in this experiment (YEASEN, Shanghai, China). Signals were detected using the Odyssey Two-color Infrared Laser Imaging System (Li-Cor, Lincoln, NE).

### 2.13. Quantitative Real-Time PCR Analysis

Total RNA from cells or tissues was extracted using the TRIzol reagent according to the manufacturer's instructions. Approximately 500 ng of extracted RNA was used to synthesize cDNA using the reverse transcription kit (TaKaRa Biotechnology, Dalian, China). Quantitative PCR was carried out in the Applied Biosystems 7500 Real-Time PCR System using 50 ng of cDNA and a SYBR Green PCR master mix (YEASEN, Shanghai, China). Relative gene expression was calculated based on 2^△△CT^ algorithm. All the primers were designed using the principle of span exons to avoid genomic DNA contamination. Primer sequences utilized in this study are provided in Table S2.

### 2.14. Statistical Analyses

Two-group comparisons were analyzed by Student's *t*-test; multigroup comparisons were analyzed via one-way ANOVA. Spearman's correlation analysis was performed to evaluate expression correlation. Kaplan-Meier analysis was carried out to analyze overall survival. Multivariate analysis was performed using the Cox multivariate regression analysis model. *P* values < 0.05 were considered statistically significant. Statistical analysis was performed with SPSS 17.0 software (IBM).

## 3. Results

### 3.1. Analysis of Gene Expression Patterns of PPARA and DNA Methyltransferase in Human Colon Cancer

**Figure 1:**
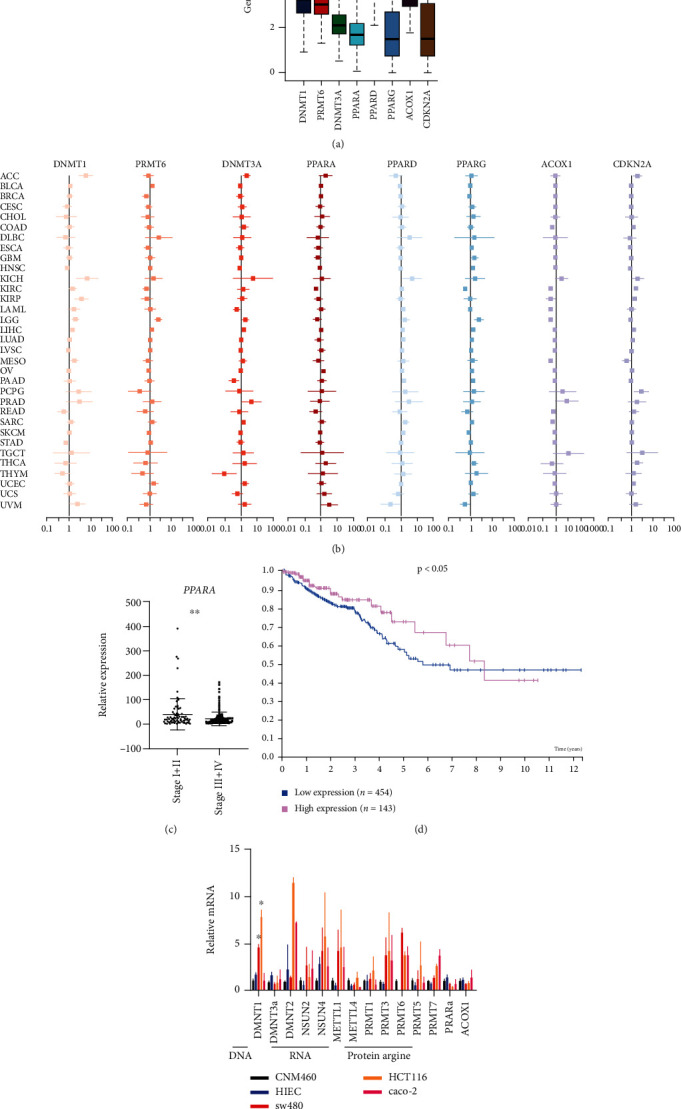
Bioinformatics analysis of PPARA in pancancer: (a) the box plot showing the expression levels of PPARs, DNMT1, PRMT6, ACOX1, and CDKN2A in tumorous tissues; (b) the forest graph showing the hazard ratio of PPARs, ACOX1, CDKN2A, methylation transferase DNMT1, DNMT3a, and PRMT6 in various cancers; (c) box plots of PPARA expression detected in RNA-seq in colon cancer specimens grouped into stage I + II and stage III + IV; (d) survival curve of patients with different PPARA protein expression levels in colon cancer (*P* < 0.05); (e) the relative mRNA expression of PPARA, ACOX1, and methylation transferases was measured using qRT-PCR in SW480, HCT116, Caco-2, HIEC, and NCM460 cell lines. The data is expressed as mean ± SD (^∗^*P* < 0.05, ^∗∗^*P* < 0.01, ^∗∗∗^*P* < 0.001).

First, we performed pancancer analyses to identify the mean expression levels of PPARs in different types of tumors. The results indicated that PPARA was lowly expressed in tumorous tissues, as shown in Figure 1(a). Subsequently, the Cox proportional hazards regression analysis was used to evaluate PPARs as prognostic markers in various tumors. Genes with a hazard ratio (HR) > 1 were significantly correlated with patient outcome. The forest plot in Figure 1(b) shows that in most cancer types, PPARs, including PPARA, may serve as a prognostic indicator of digestive tract cancers. Subsequently, we focused on the clinical significance of PPARA expression. PPARA was downregulated in colon cancer and correlated with TNM stage in the TCGA COAD dataset (Figure 1(c)). Furthermore, following the evaluation of PPARA protein levels, the prognosis of patients with high PPARA or low PPARA expression revealed that higher expression exhibited a better prognosis (Figure 1(d)).

Recent reports have demonstrated that DNA hypermethylation of tumor suppressor genes contributes to cancer progression. In the current study, the mean expression levels of the DNA methyltransferases DNMT1 and DNMT3a and the protein arginine methyltransferase, PRMT6, were evaluated in pancancers using bioinformatics analyses. High levels of several methyltransferases were observed in various tumor tissues (Figure 1(a)). The forest graph indicated the prognostic value of DNMT1 in most cancers, with an HR > 1 (Figure 1(b)). In addition, colon cancer cell lines with high endogenous expression of methyltransferases and low endogenous expression of PPARA relative to normal NCM460 and HIEC cells were selected for further *in vitro* assays (Figure 1(e)).

### 3.2. The PPARA Agonist, Fenofibrate, Attenuated Cell Viability and Proliferation

**Figure 2:**
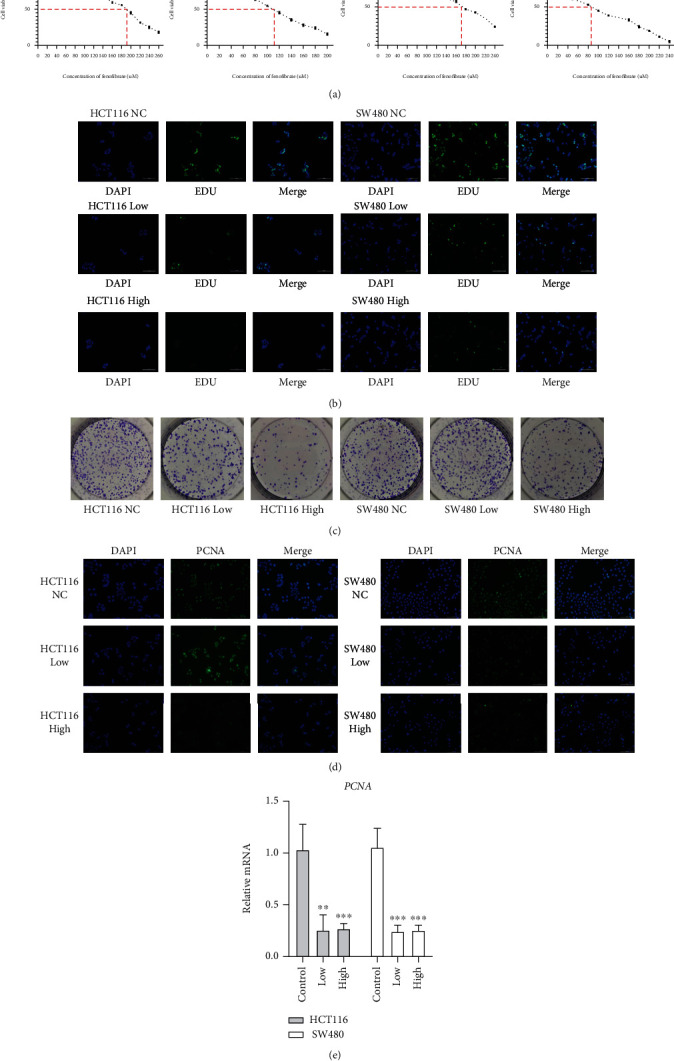
Fenofibrate administration inhibited colon cancer cell proliferation. (a) HCT116 and SW480 cells were treated with a range of concentrations of fenofibrate for 24 h. Cell viability was detected using CCK8. (b) EDU staining of cells was observed after incubation with fenofibrate for 24 h (magnification 200x). Box plot showing the statistics of fluorescence intensity. (c) Colony formation of cancer cells with or without fenofibrate treatment. (d) The PCNA immunofluorescence staining results of cells (magnification 200x). (e) The mRNA expression level of PCNA was measured following fenofibrate treatment. Data was presented as mean ± SD. The experiment was repeated three times with three replicates per experiment (^∗^*P* < 0.05, ^∗∗^*P* < 0.01, ^∗∗∗^*P* < 0.001).

The effect of the PPAR agonist on colon cancer cell proliferation was assessed by treating 5000 cells with various concentrations of fenofibrate (0–300 *μ*mol/L) for 24 and 48 h. Cell survival was determined using the CCK8 kit. As shown in Figure 2(a), fenofibrate exerted an inhibitory effect on cell proliferation in HCT116 cells with an IC50 range of 180–200 *μ*mol/L (24 h) and 100–120 *μ*mol/L (48 h); fenofibrate also exerted an inhibitory effect on cell proliferation in SW480 cells, with an IC50 range of 160–180 *μ*mol/L (24 h) and 80–100 *μ*mol/L (48 h).

We selected suitable concentrations of fenofibrate (180 and 200 *μ*mol/L for HCT116 cells and 160 and 180 *μ*mol/L for SW480 cells) for subsequent treatments. After incubation with fenofibrate for 24 h, cells were stained with EDU (green) and DAPI (blue). Treatment of fenofibrate decreased the proportion of cells with green fluorescence in a dose-dependent manner (*P* < 0.05) (Figure 2(b)). To further demonstrate the inhibitory effect of fenofibrate on cell growth, the colony formation assay was employed. Treatment of HCT116 and SW480 cells with different doses of fenofibrate reduced clone numbers, especially in the high-dose group (Figure 2(c)). The expression level of PCNA, an endogenous marker of mitogenesis, was detected between the low-dose, high-dose, and vehicle-treated groups using qRT-PCR and immunofluorescence staining. Reduced PCNA mRNA level and protein level upon fenofibrate treatment suggested the suppressive regulation of cell proliferation by fenofibrate (Figures 2(d) and 2(e)).

### 3.3. The PPAR Agonist, Fenofibrate, Promoted Cell Apoptosis *In Vitro*

**Figure 3:**
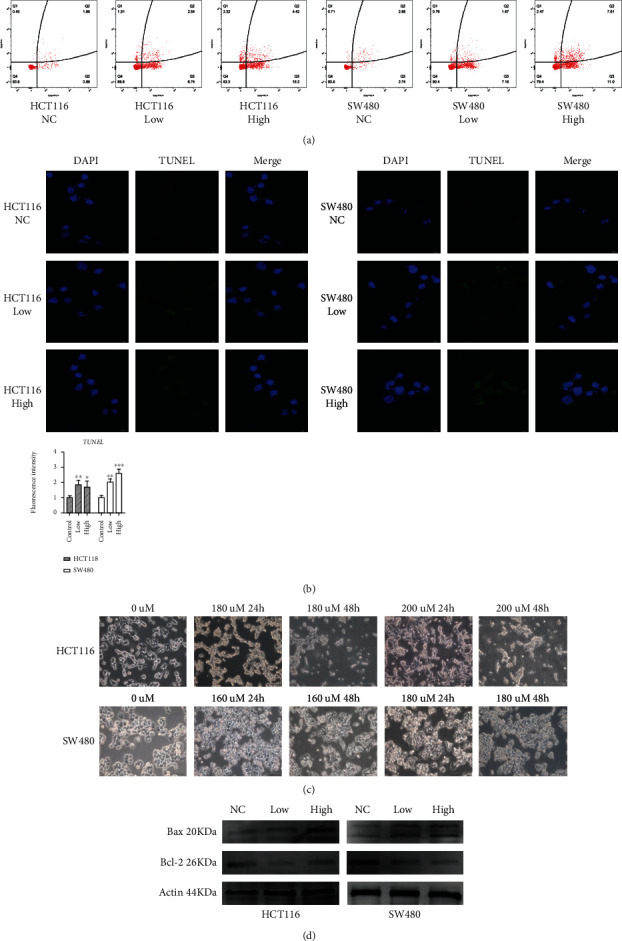
Fenofibrate treatment promoted colon cancer cell apoptosis. (a) Cell apoptosis was analyzed using flow cytometry. (b) TUNEL staining of colon cancer cells was observed after treatment of fenofibrate for 24 h. TUNEL-stained (green) cells indicate apoptosis-positive cells, DAPI (blue) indicates nucleated cells, and the merge column shows cells stained with TUNEL and DAPI. (c) Morphological changes were observed of HCT116 and SW480 cells after treatment of fenofibrate for 24 h. (d) The protein expression of Bax and Bcl-2 in SW480 and HCT116 cells was measured using western blot.

To investigate whether PPARA activation promoted cell apoptosis *in vitro*, colon cancer cells with or without fenofibrate treatment were assessed by flow cytometry. The data suggested that fenofibrate increased cell apoptosis and, in particular, late apoptosis rates (Figure 3(a)). Subsequently, we confirmed the proapoptotic effects of fenofibrate using TUNEL staining, which detects the DNA breaks in apoptotic cells.  Figure 3(b) shows that fenofibrate administration increased the number of positively stained cells (green fluorescence). Moreover, the changes in cell shape after incubation with fenofibrate was examined using light microscopy. The number of shedding cells was increased, accompanied with morphological deformation and crumpled appearances. Such morphological changes were observed in a time- and dose-dependent manner (Figure 3(c)). The expression level of Bax, a typical apoptosis-related protein which promotes apoptosis was increased by treatment with fenofibrate, while prosurvival protein Bcl-2 was decreased as shown by western blotting (Figure 3(d)).

### 3.4. The PPAR Agonist, Fenofibrate, Inhibited Cell Migration and Epithelial Mesenchymal Transition

**Figure 4:**
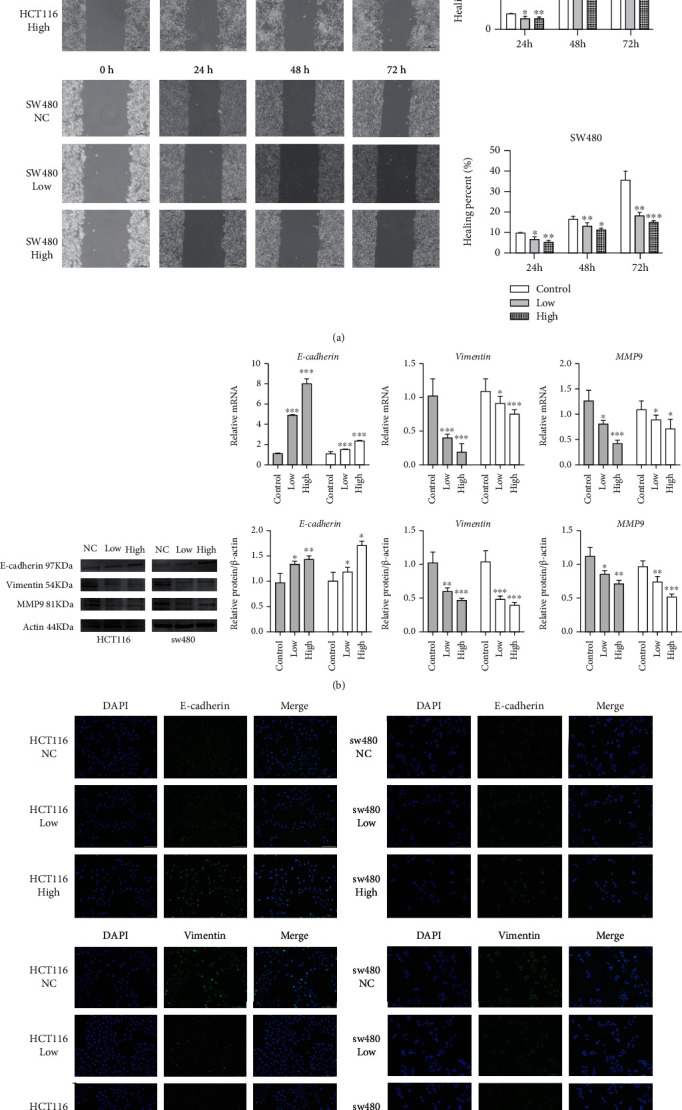
Fenofibrate inhibited cell migration and EMT. (a) Wound healing assay for demonstrating the inhibitory effect of fenofibrate on the migration of colon cancer cells at 0, 24, 48, and 72 h following wounding. (b) The mRNA and protein expression levels of E-cadherin, vimentin, and MMP9 were measured following fenofibrate treatment. Data was presented as mean ± SD (^∗^*P* < 0.05, ^∗∗^*P* < 0.01, ^∗∗∗^*P* < 0.001). (c) The expression of E-cadherin and vimentin in colon cancer cells was examined using immunofluorescence staining (magnification 200x).

Cell migration was measured using the wound healing assay in fenofibrate-treated cells. Data from the scratch healing assay are shown in Figure 4(a). Fenofibrate significantly reduced the migratory capacity of CRC cells, which exhibited delays in the closure of scratches. Next, we examined whether there was a repressive effect of PPARA activation on epithelial mesenchymal transition (EMT). The expression of several EMT biomarkers of EMT was tested using qRT-PCR (Figure 4(b)). The mRNA levels of vimentin and MMP9 in the fenofibrate-treated group were reduced, while E-cadherin levels were upregulated. To further confirm the findings, expression changes in the EMT-associated markers induced by fenofibrate were examined using immunofluorescence, and similar results were observed (Figure 4(c)). Taken together, PPARA activation could exert an antitumor effect by restraining cell migration and EMT.

### 3.5. The PPAR Agonist, Fenofibrate, Enhanced Tumor Suppressor Gene Expression and Repressed DNMT1 Content

**Figure 5:**
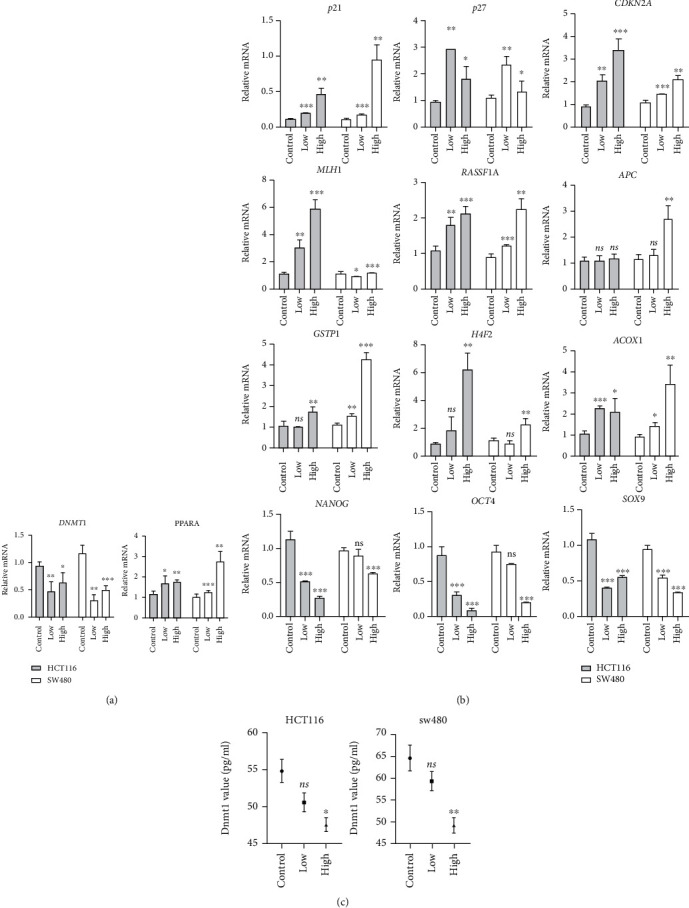
Fenofibrate decreased the content of DNMT1 and increased the expression of tumor suppressor genes. (a) qRT-PCR analysis of DNMT1 and PPARA mRNA expression in HCT116 and SW480 cells following fenofibrate treatment. Data was presented as mean ± SD (^∗^*P* < 0.05, ^∗∗^*P* < 0.01, ^∗∗∗^*P* < 0.001). (b) The mRNA expression of p21, p27, CDKN2A, MLH1, RASSF1A, APC, GSTP1, H4F2, ACOX1, Oct4, Nanog, and Sox9 was measured. Data was presented as mean ± SD (^∗^*P* < 0.05, ^∗∗^*P* < 0.01, ^∗∗∗^*P* < 0.001). (c) DMNT1 value was measured using ELISA kit. Data was presented as mean ± SD (^∗^*P* < 0.05, ^∗∗^*P* < 0.01, ^∗∗∗^*P* < 0.001).

The research of Luo et al. [28] substantiated that the loss of PPARA resulted in abnormal expression of several methyltransferases and promoted CRC progression in a mouse model. Therefore, we hypothesized that the PPARA agonist, fenofibrate, could reduce the content of DNMT1 and rescue the expression of methylation-silenced tumor suppressor genes. First, we found using qRT-PCR that the DNMT1 mRNA level decreased when PPARA was activated following fenofibrate treatment (Figure 5(a)).

Next, the expression of several tumor suppressor genes was determined (Figure 5(b)). It has been documented that the expression of silenced genes is correlated with promoter hypermethylation. Studies showed that p21, p27, CDKN2A, MLH1, and RASSF1A were reactivated in two cancer cell lines after fenofibrate treatment. We further discovered that the expression of the upstream regulators of DNMT1, Oct4, Nanog, and Sox9 was decreased in fenofibrate-treated cells and were correlated with stemness. In addition, we measured the DNMT1 enzyme content in colon cancer cells and found a significant reduction in enzyme concentration upon high-dose treatment of fenofibrate (Figure 5(c)).

### 3.6. Fenofibrate Recovered the Expression of CDKN2A via Downregulation of DNMT1

**Figure 6:**
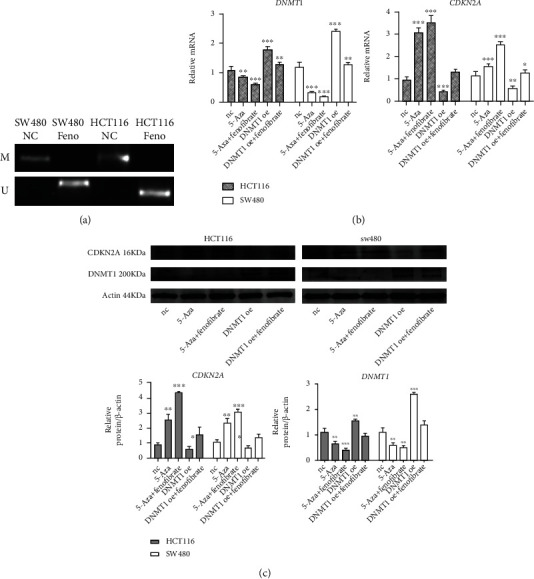
DNMT1 repressed CDKN2A expression by promoter hypermethylation. (a) MSP results showed the methylation status of CDKN2A following fenofibrate treatment. (M: reactions using CDKN2A primers specific for methylated CpG sites; U: reactions using CDKN2A primers specific for unmethylated CpG sites). (b) The mRNA expression of DNMT1 and CDKN2A in cells was measured using qRT-PCR. Data was presented as mean ± SD (NC: untreated cell; 5-Aza: 5-azacytidine treated cell; DNMT1 oe: DNMT1 overexpression cell; ^∗^*P* < 0.05, ^∗∗^*P* < 0.01, ^∗∗∗^*P* < 0.001). (c) The protein expression of CDKN2A and DNMT1 in SW480 and HCT116 cells was measured using western blot.

To determine the mechanisms of fenofibrate treatment on the demethylation of the CDKN2A promoter, MSP was carried out to evaluate the methylation status of the CDKN2A promoter. In the Figure 6(a), M and U referred to the PCR products of methylated and unmethylated alleles, respectively. Analyses showed that in the fenofibrate-free group, a methylation product was observed, while no band was observed in the fenofibrate-treated group. Thus, we inferred that the methylation status of the promoter was abolished by fenofibrate treatment (Figure 6(a)).

5-Azacytidine is an effective inhibitor of DNMT1. To investigate the effects of the overexpression or downexpression of DNMT1 on CDKN2A, colon cancer cells were treated with 5-azacytidine, DNMT1 overexpression plasmid, and fenofibrate. The results showed that DNMT1 downexpression resulted in increased CDKN2A mRNA and protein levels, while DNMT1 overexpression caused a decreased level of CDKN2A (Figures 6(b) and 6(c)). The expression changes of CDKN2A induced by 5-azacytidine could be enhanced by fenofibrate treatment. In addition, fenofibrate could reverse the low expression of CDKN2A caused by DNMT1 overexpression plasmid. Taken together, these findings indicated that the PPARA agonist, fenofibrate, could upregulate the expression of CDKN2A by inhibiting gene hypermethylation mediated by DNMT1.

### 3.7. Fenofibrate Regulates the Cell Cycle via the CDKN2A/RB1/E2F1 Pathway

**Figure 7:**
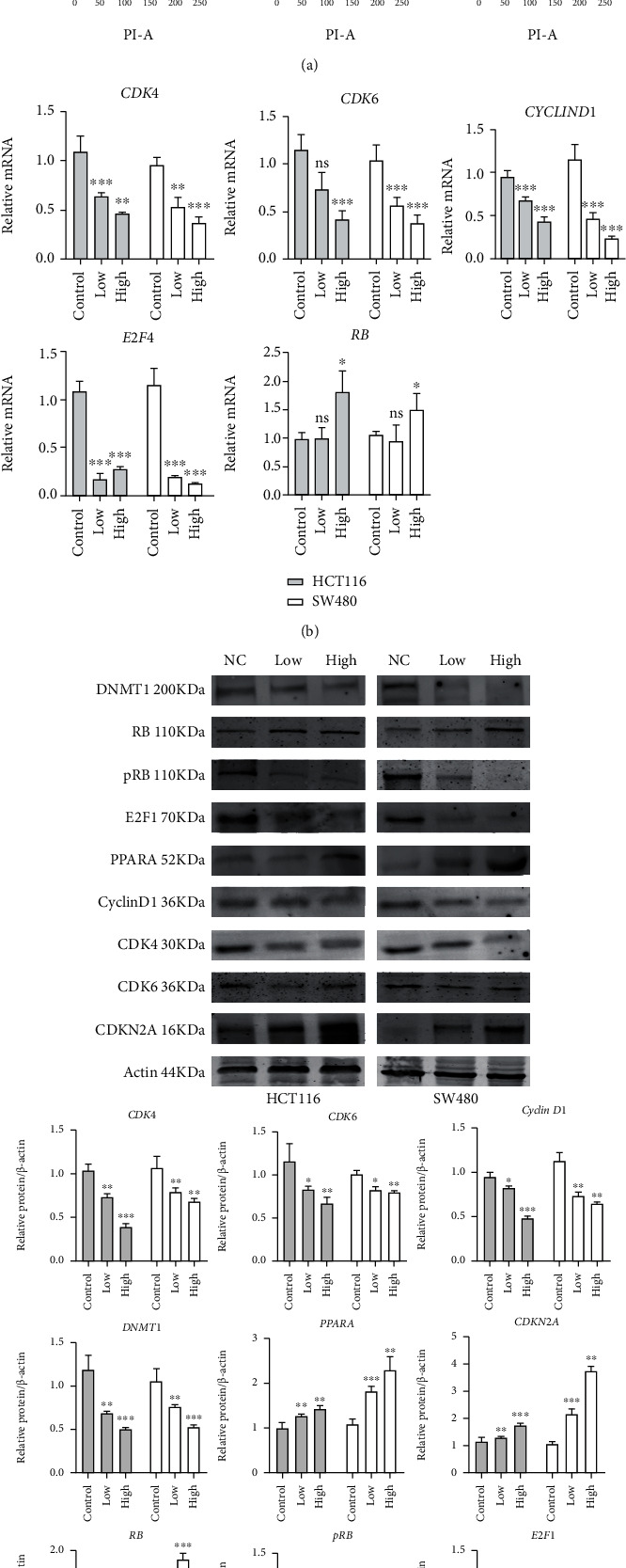
Fenofibrate modulated cell cycle via CDKN2A/RB/E2F transcript cascade. (a) Cell cycle distribution was examined using flow cytometry. (b) The mRNA expression of RB, E2F1, CDK4, CDK6, and Cyclin D1 was measured using qRT-PCR. Data was presented as mean ± SD (^∗^*P* < 0.05, ^∗∗^*P* < 0.01, ^∗∗∗^*P* < 0.001). (c) Western blot analysis of DNMT1, RB, pRB, E2F1, PPARA, Cyclin D1, CDK4, CDK6, and CDKN2A expression in colon cancer cells.

DNMT1 is responsible for maintaining DNA methylation after each round of the cell cycle. CDKN2A, a CDK inhibitor, acts as a negative regulator of cell cycle process. In the current study, fenofibrate administration suppressed the expression of DNMT1 and relieved the DNMT1-mediated silencing of CDKN2A. Therefore, we investigated the molecular mechanism by which fenofibrate regulated cell cycle distribution. PI staining was conducted on control and drug-treated cells, and the results of cell cycle analysis are shown in Figure 7(a). Fenofibrate treatment suppressed the G1→S transition, induced G0/G1 phase cell arrest, and blocked S phase entry. Since the activity of the cyclin D/CDK4/CDK6 complex is essential to the G1/S transition and can be inhibited by CDK inhibitors, we detected the changes in expression of cyclinD1, CDK4, and CDK6 in cells following fenofibrate treatment. The results from western blot and qRT-PCR analyses suggested that fenofibrate induced the activation of CDKN2A, resulting in low expression levels of CDKs (Figures 7(b) and 7(c)). During the G1 phase, the level of E2F1 was upregulated by the activated cyclin-CDK complex and was released from the RB/E2F contigs in response to abnormal growth stimulation.

To further validate our findings, the expression of key factors involved in the RB/E2F signaling pathway was examined. The expression of total RB was upregulated, while the levels of phosphorylated RB and E2F1 were reduced following fenofibrate treatment. These results demonstrated that fenofibrate treatment decreased the protein and transcript levels of pRB and E2F through the downregulation of cyclin-CDKs by activated CDKN2A.

### 3.8. The PPAR Agonist, Fenofibrate, Inhibited Tumor Growth in an Animal Model

We established an animal model of HCT116 cell-bearing nude mice. When tumor tissues reached an average volume of 1 mm^3^, mice were randomized into two groups. Mice in the treatment group received 200 mg/kg fenofibrate suspended in 200 *μ*L saline by gavage every day. The control group was gavaged with an equal volume of saline. All mice bearing tumors survived during the experiment. Tumor size was significantly smaller in the drug-treated group compared to the cancer group.

**Figure 8:**
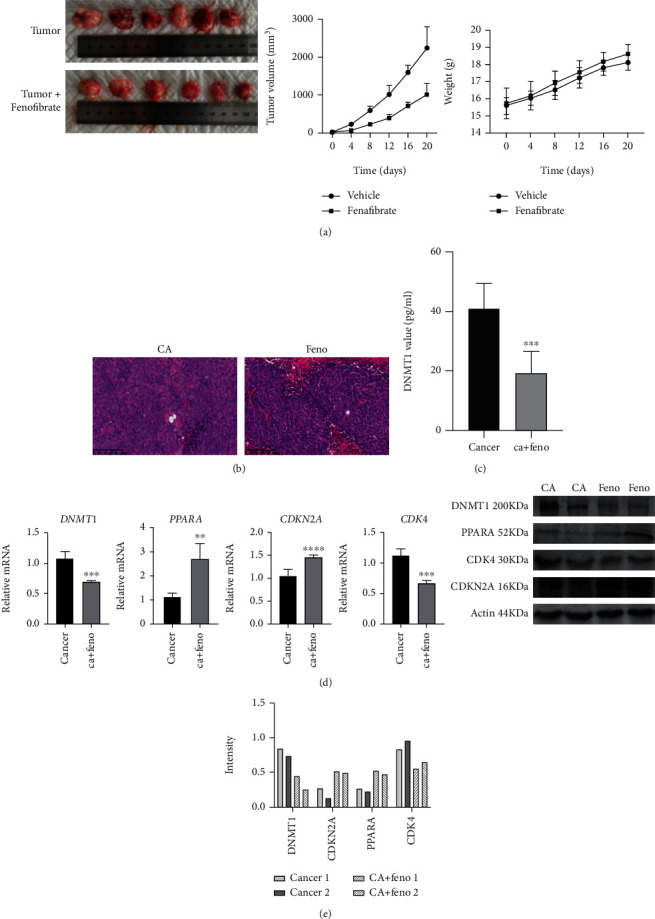
Fenofibrate suppressed tumor growth and DNMT1 content *in vivo*. (a) Representative images of subcutaneous xenografts from the treatment and vehicle groups (*n* = 6 mice per group). Subcutaneous xenograft growth curves of nude mice of the two groups. Body weight of each mouse was recorded every three days. Data was presented as mean ± SD. (b) H&E staining of subcutaneous xenografts from the treatment group and vehicle group (magnification 200x). (c) The DNMT1 value of tissues was examined using ELISA assay. Data was presented as mean ± SD (^∗^*P* < 0.05, ^∗∗^*P* < 0.01, ^∗∗∗^*P* < 0.001). (d) DNMT1, PPARA, CDK4, and CDKN2A mRNA expressions of xenografts from two groups were detected using qRT-PCR. Data was shown as mean ± SD (^∗^*P* < 0.05, ^∗∗^*P* < 0.01, ^∗∗∗^*P* < 0.001). (e) DNMT1, PPARA, CDK4, and CDKN2A protein levels in different groups were measured by western blot.

The weight of each mouse was recorded twice weekly, and the data are shown in Figure 8(a). Tumor xenografts were sectioned and stained with H&E to observe the pathological changes. Slides from fenofibrate-treated tissues revealed necrotic lesions (Figure 8(b)). DMNT1 content in tissue homogenates was measured by an ELISA assay. Following fenofibrate treatment, DNMT1 enzyme level in tissues from the treatment group was lower than that from the vehicle group (Figure 8(c)). Additionally, fenofibrate significantly downregulated the mRNA and protein levels of DNMT1 and CDK4 and increased the expression levels of PPARA and CDKN2A (Figures 8(d) and 8(e)). Taken together, PPARA activation suppressed tumors *in vivo* by upregulating the expression of CDKN2A.

## 4. Discussion

The role of PPARA in tumor initiation and development remains controversial. Several studies have elucidated the antitumor effect of PPARA in various cancers, including breast cancer, prostate cancer, and ovarian cancer [32–34]. Some studies have drawn contradictory conclusions, suggesting that prolonged administration of PPARA agonists might cause hepatocarcinogenesis; however, the detailed mechanism remains unclear [35]. In the present study, we analyzed the expression levels of PPARs in pancancers and noted that PPARA was expressed at low levels in several types of tumors, including colon cancer. Colon cancer samples with high PPARA protein expression were observed to have a better prognosis than those with low PPARA levels. These results suggested that PPARA might serve as a tumor suppressor gene in colon cancer. Most recently, PPARA-specific agonists were reported to exhibit anticancer effects in a variety of tumors. Fenofibrate is a PPARA activator that belongs to the fibrate class of drugs. An increasing number of studies have revealed its potential role as an antitumor agent that affects multiple biological pathways [36–39].

DNMT1 is responsible for maintaining global methylation and aberrant CGI methylation in human cancer cells, whereas DNMT3a and DNMT3b are believed to act as maintenance and de novo methyltransferases. The elevated expression of DNMT1 has been reported in colon adenocarcinomas, hepatocarcinomas, and lung cancer [40–42].

Hypermethylation of gene promoter regions leads to transcriptional repression. Tse et al. [4] showed that promoter methylation of tumor suppressor genes promoted carcinogenesis of colon cancer. In this study, we observed that fenofibrate treatment increased PPARA expression and decreased DNMT1 activity, accompanied with the elevated expression of a series of established tumor suppressor genes, including RASSF1A, MLH1, p21, and p27. CDKN2A mRNA and protein levels were upregulated in both HCT116 and SW480 fenofibrate-treated cells compared to controls. To confirm the hypothesis that fenofibrate abrogated the hypermethylation of CDKN2A, we detected the methylation status of its promoter using methylation-specific PCR. In reactions using methylation-specific primers, no band for methylated CDKN2A was observed in the fenofibrate-treated group. Furthermore, the expression levels of DNMT1 and CDKN2A were measured in colon cancer cells following treatment with the DNMT1 inhibitor, 5-azacytidine, and DNMT1 overexpression plasmid. The results indicated that fenofibrate functions as a repressor, similar to a methyltransferase inhibitor.

There are several reports suggesting that PPARA activation inhibits cell proliferation by targeting the cyclin-dependent kinase inhibitor, CDKN2A [43, 44]. In the present study, we found that cells treated with fenofibrate were arrested in the G1 phase, and the number of G2/M cells was reduced. Moreover, fenofibrate elevated the level of CDKN2A by suppressing DNMT1 expression, reduced the activation of cyclin D1/CDK complexes, and phosphorylated RB. Cyclin D1/CDK complexes are cell cycle-related molecules that facilitate the G1/S transition [45]. In quiescent cells, hypophosphorylated RB protein interacts with E2F and inhibits its transcription activity. Under growth stimulation or cancerous states, cyclin-CDK complexes are activated to induce RB phosphorylation. Phosphorylated RB then releases the E2F transcription factor, which triggers the transition of the cell cycle from the G1 phase to the S phase, thereby, enabling uncontrolled cell proliferation [46]. In summary, the RB/E2F pathway was involved in fenofibrate-mediated epigenetic changes on CDKN2A, which resulted in the alterations in cell cycle distribution.

Some studies have shown that DNMT1 cooperated directly with E2F1 and HDAC to accelerate aberrant methylation in tumors [47]. The free E2F1 is released by phosphorylated RB and binds to its cognate sites on the DNMT1 promoter region, which played a positive role on DNMT1 expression in cell cycle process [48]. Elevated expression of DNMT1 induced DNA hypermethylation of several tumor suppressor genes, including CDKN2A. However, conflicting results have emerged regarding the relationship between DNMT1 and E2F1. In mesenchymal stem cells, the expression of E2F1 was not correlated with that of DNMT1. Complete cell cycle arrest by serum starvation did not affect the expression of DNMT1, while E2F1 expression was decreased [49]. The above findings revealed that DNMT1 may not be a responsive target of E2F1 during cell cycle arrest.

In the present study, we found that fenofibrate may act in a similar manner as a methylation transferase inhibitor. It reduced DNMT1 activity and E2F1 expression. The mechanistic details behind PPAR agonist on DNMT1 inhibition have not been determined. Whether DNMT1 downregulation caused decreases in free E2F1 requires further investigation.

In addition, we demonstrated that fenofibrate inhibited tumor progression by regulating cell apoptosis and migration. The results from flow cytometry analysis and TUNEL assays showed that fenofibrate caused an increase in late apoptosis in a dose-dependent manner. However, the underlying mechanism for such findings was not determined in our study.

Cellular plasticity mediated by EMT regulatory circuits enhances the invasive properties of cancer cells [50]. Transcriptional repression of E-cadherin is frequently observed in malignant tumor cells. Some studies have verified that DNMT1 caused the suppression of E-cadherin through hypermethylation of its promoter region [51]. We found that E-cadherin expression was increased following fenofibrate treatment *in vitro*; however, whether the upregulation was correlated with reduced DNMT1 activity and promoter demethylation requires further investigation.

Finally, we carried out tumor xenograft experiments using HCT116 cells to investigate the antitumor efficacy of fenofibrate *in vivo*. Fenofibrate decreased the tumor volume significantly compared to the vehicle-treated mice. The necrotic area was identified in H&E-stained samples from fenofibrate-treated mice. The expression of DNMT1, CDK4, and CDKN2A was effectively reduced by fenofibrate treatment compared with control cells. These results demonstrated that fenofibrate could ablate tumors and retard tumor growth.

These findings, coupled with the reversibility of DNA methylation, support the possibility of fenofibrate as a potential epigenetic treatment in colon cancer patients.

## 5. Conclusions

In conclusion, the present work illustrated that activation of PPARA by fenofibrate administration protected against colon cancer progression through epigenetic modifications. Fenofibrate weakened DNMT1 activity and restored the expression of the tumor suppressor gene, CDKN2A, which suppressed cell proliferation by blocking the G1 to S transition through the RB/E2F pathway. In addition, fenofibrate inhibited cancer cell invasion by regulating EMT. Therefore, we conclude that fenofibrate could act as an adjuvant agent in colon cancer treatment.

## Data Availability

TCGA gene expression data and clinical data were obtained from TCGA data portal (https://portal.gdc.cancer.gov).

## Abbreviations

ACC: Adrenocortical carcinoma
BLCA: Bladder urothelial carcinoma
BRCA: Breast invasive carcinoma
CESC: Cervical squamous cell carcinoma and endocervical adenocarcinoma
CHOL: Cholangiocarcinoma
COAD: Colon adenocarcinoma
DLBC: Lymphoid neoplasm diffuse large B-cell lymphoma
ESCA: Esophageal carcinoma
GBM: Glioblastoma multiforme
HNSC: Head and neck squamous cell carcinoma
KICH: Kidney chromophobe
KIRC: Kidney renal clear cell carcinoma
KIRP: Kidney renal papillary cell carcinoma
AML: Acute myeloid leukemia
LGG: Brain lower grade glioma
LIHC: Liver hepatocellular carcinoma
LUAD: Lung adenocarcinoma
LUSC: Lung squamous cell carcinoma
MESO: Mesothelioma
OV: Ovarian serous cystadenocarcinoma
PAAD: Pancreatic adenocarcinoma
PCPG: Pheochromocytoma and paraganglioma
PRAD: Prostate adenocarcinoma
READ: Rectum adenocarcinoma
SARC: Sarcoma
SKCM: Skin cutaneous melanoma
STAD: Stomach adenocarcinoma
TGCT: Testicular germ cell tumors
THCA: Thyroid carcinoma
THYM: Thymoma
UCEC: Uterine corpus endometrial carcinoma
UCS: Uterine carcinosarcoma
UVM: Uveal melanoma
FPKM: Fragments per kilobase per million
TCGA: The Cancer Genome Atlas
CGI: CpG islands
CRC: Colorectal cancer
PPARA: Peroxisome proliferator-activated receptor alpha
DNMT1: DNA methyltransferase 1
RB: Retinoblastoma
EMT: Epithelial mesenchymal transition
HR: Hazard ratio
qRT-PCR: Quantitative reverse transcription PCR
MSP: Methylation-specific PCR
EdU: 5-Ethynyl-2′-deoxyuridine
PI: Propidium iodide
SDS: Sodium dodecylsulfate-polyacrylamide.

## Supplementary Materials

Supplementary Materials Table S1: list of primers used in the qRT-PCR assay. Table S2: list of primers used in the methylation-specific PCR assay.
