# Supplementary material for: Fenofibrate Exerts Antitumor Effects in Colon Cancer via Regulation of DNMT1 and CDKN2A
Source: PPAR Res. 2021 Apr 16;2021:6663782. doi: 10.1155/2021/6663782 (PMC8075693; doi:10.1155/2021/6663782)
Supplement: Supplementary Materials — Table S1: list of primers used in the qRT-PCR assay. Table S2: list of primers used in the methylation-specific PCR assay. [file 6663782.f1.pdf]

Table S1 Primer Sequence used in qRT-PCR (5'-3')

Gene	Forward	Reverse
M Ppara	CCCTGAACATCGAGTGTCGAA	TTCGCCGAAAGAAGCCCTTA
M Dnmt1	TGTTCTGTCGTCTGCAACCT	GCCATCTCTTTCCAAGTCTTT
M Cdk4	CTGGAAGAAGTCTGCGTCGG	GTCTTGCCAAAGCGGTTCAG
M CDKN2A	GCGAGACGGACAAACACTG	ACTTTGATGCCTTCAGCCATAC
M β-actin	GGCTGTATTCCCCTCCATCG	CCAGTTGGTAACAATGCCATGT
H PPARA	TCATCACGGACACGCTTTCA	TCAATGCTCCACTGGGAGAC
H DNMT1	CCTAGCCCCAGGATTACAAGG	ACTCATCCGATTTGGCTCTTTC
H PRMT6	GGAGTCGGAGAAACCCCTG	TGAAACGTCCGTGTCTTGCTC
H P21	CGATGGAACTTCGACTTTGTCA	GCACAAGGGTACAAGACAGTG
H P27	GTACGAGTGGCAAGAGGTGG	TAGAAGAATCGTCGGTTGCAGG
H ACTIN	CATGTACGTTGCTATCCAGGC	CTCCTTAATGTCACGCACGAT
H DNMT3A	CCGATGCTGGGGACAAGAAT	CCCGTCATCCACCAAGACAC
H DNMT2	CGGGTGCTGGAGCTATACAG	CGACAGTGTTGACATCAATGGC
H ACOX1	ACTCGCAGCCAGCGTTATG	AGGGTCAGCGATGCCAAAC
H NSUN2	CAAGCTGTTCGAGCACTACTAC	CTCCCTGAGAGCGTCCATGA
H NSUN4	CCATCAATCCGTGTCAGTCTC	GCTTAGCACTTACATGATCCCAG
H METTL1	GGCAACGTGCTCACTCCAA	CACAGCCTATGTCTGCAAACT
H METTL3	TTGTCTCCAACCTTCCGTAGT	CCAGATCAGAGAGGTGGTGTAG
H PRMT1	CTTTGACTCCTACGCACACTT	GTGCCGGTTATGAAACATGGA
H PRMT3	GTACCCTTCTCATACCCCAATGG	GACGAGCAGGTTCTGACATCT
H PRMT5	CTGTCTTCCATCCGCGTTTCA	GCAGTAGGTCTGATCGTGTCTG
H PRMT6	TACCGCCTGGGTATCCTTCG	CCTGTTCCGGCAACTCTACA
H PRMT7	TTGACACAGAGCTGATCGGG	CAACGGGAGGGACGATGAC
H PRMT8	CCTGCTAAGCCCGTGCAAT	TGGGCATAGGAGTCGAAGTAA
H PCNA	GCTGACATCGGACACTTA	CTCAGGTACAAACTTGGTG
H MMP9	TGTACCGCTATGGTTACACTCG	GGCAGGGACAGTTGCTTCT
H VIMENTIN	GACGCCATCAACACCGAGTT	CTTTGTCGTTGGTTAGCTGGT
H ECADHERIN	CGAGAGCTACACGTTCACGG	GGGTGTCGAGGGAAAAATAGG
H CDKN2A	GATCCAGGTGGGTAGAAGGTC	CCCCTGCAAACTTCGTCCT
H DNMT3B	AGGGAAGACTCGATCCTCGTC	GTGTGTAGCTTAGCAGACTGG
H MLH1	CTCTTCATCAACCATCGTCTGG	GCAAATAGGCTGCATACACTGTT
H H4F2	GAAAAGGCTTAGGCAAAGGGG	CCAGAGATCCGCTTAACGC
H RASSF1A	AGGACGGTTCTTACACAGGCT	TGGGCAGGTAAAAGGAAGTGC
H APC	AAAATGTCCCTCCGTTCTTATGG	CTGAAGTTGAGCGTAATACCAGT
H GSTP1	CCCTACACCGTGGTCTATTTCC	CAGGAGGCTTTGAGTGAGC
H CyclinD1	GCTGCGAAGTGGAAACCATC	CCTCCTTCTGCACACATTTGAA
H CDK4	ATGGCTACCTCTCGATATGAGC	CATTGGGGACTCTCACACTCT
H CDK6	GCTGACCAGCAGTACGAATG	GCACACATCAAACAACCTGACC
H RB1	CTCTCGTCAGGCTTGAGTTTG	GACATCTCATCTAGGTCAACTGC
H OCT4	CTGGGTTGATCCTCGGACCT	CCATCGGAGTTGCTCTCCA
H NANOG	TTTGTGGGCCTGAAGAAAACT	AGGGCTGTCCTGAATAAGCAG
H SOX2	GCCGAGTGGAAACTTTTGTCG	GGCAGCGTGTACTTATCCTTCT

H SOX9	AGCGAACGCACATCAAGAC	CTGTAGGCGATCTGTTGGGG
H HIC1	GTCGTGCGACAAGAGCTACAA	CGTTGCTGTGCGAACTTGC
H E2F	ACGCTATGAGACCTCACTGAA	TCCTGGGTCAACCCCTCAAG

Table S2 Primer Sequence used in MSP (5'-3')

Gene	Forward	Reverse
CDKN2A M	TTATTAGAGGGTGGGGCGGATCGC	GACCCCGAACCGCGACCGTAA
CDKN2A U	GAGGGGTTGGTTGGTTATTAG	CCACCTAAATCAACCTCCAACCA
